# Anti-Inflammatory Effects of *Lagerstroemia ovalifolia* Teijsm. & Binn. in TNF*α*/IFN*γ*-Stimulated Keratinocytes

**DOI:** 10.1155/2021/2439231

**Published:** 2021-11-09

**Authors:** Han-Sol Lee, Jin-Hyub Paik, Ok-Kyoung Kwon, Imam Paryanto, Prasetyawan Yuniato, Hyung Won Ryu, Sang-Ho Choi, Sei-Ryang Oh, Sang-Bae Han, Ji-Won Park, Kyung-Seop Ahn

**Affiliations:** ^1^Natural Medicine Research Center, Korea Research Institute of Bioscience and Biotechnology, 30 Yeongudanji-ro, Ochang-eup, Cheongwon-gu, Cheongju-si, Chungcheongbuk-do 28116, Republic of Korea; ^2^College of Pharmacy, Chungbuk National University, Osongsaengmyeong 1-ro, Osong-eup, Heungdeok-gu, Cheongju-si, Chungcheongbuk-do 28160, Republic of Korea; ^3^International Biological Material Research Center, Korea Research Institute of Bioscience and Biotechnology, 111 Gwahangno, Yuseong-gu, Daejeon 305-806, Republic of Korea; ^4^Center for Pharmaceutical and Medical Technology, Deputy for Agroindustrial Technology and Biotechnology, The Agency for the Assessment and Application of Technology (BPPT), Jalan M.H. Thamrin, No. 8, Jakarta 10340, Indonesia

## Abstract

*Ethnopharmacological Relevance*. Atopic dermatitis is a chronic inflammatory skin disease. *Lagerstroemia ovalifolia* Teijsm. & Binn. (LO) has traditionally been used as an herbal medicine for anti-inflammatory diseases. The effect of LO on atopic dermatitis has not been verified scientifically. We investigated the effects of CHCl_3_ fraction number 5 of LO (LOC) on atopic dermatitis through cell-based experiments. HaCaT cells were treated with tumor necrosis factor-alpha (TNF*α*)/interferon-gamma (IFN*γ*) to induce an inflammatory reaction. Proinflammatory cytokines, interleukin- (IL-) 6, IL-8, and IL-1*β* and chemokines such as thymus and activation-regulated chemokine (TARC/CCL17), monocyte chemoattractant protein 1 (MCP1/CCL2), and macrophage-derived chemokine (MDC/CCL22) were measured by RT-PCR and ELISA. In addition, the degree of phosphorylation and activation of JAK/STAT1, PI3K/AKT, and nuclear factor-kappa B (NF-*κ*B) were measured by western blot and luciferase assays. The production of inflammatory cytokines and chemokines and activation of the JAK/STAT1, PI3K/AKT, and NF-*κ*B pathways were induced by TNF*α*/IFN*γ* in HaCaT cells. Under these conditions, LOC treatment inhibited the production of targeted cytokines and chemokines and decreased the phosphorylation and activation of JAK/STAT1, PI3K/AKT, and NF-*κ*B. These results suggest that LOC reduces the production of proinflammatory cytokines and chemokines by suppressing the JAK/STAT1, PI3K/AKT, and NF-*κ*B pathways. Therefore, LOC may have potential as a drug for atopic dermatitis.

## 1. Introduction

Atopic dermatitis (AD) is a chronic, recurrent inflammatory skin disease [1, 2]. This disease occurs mainly in children [3, 4]. The pathogenesis of atopy varies, with mechanisms such as allergen infiltration, an imbalance in the immune response, collapse of the skin barrier, and genetic causes [2, 5]. Therefore, this disease is difficult to treat according to the exact cause in the patient [6]. Symptoms of lesions include erythema, pus, redness, itching, epidermal hyperplasia, and lichenified skin [2, 7]. These acute and chronic lesions are mainly caused by T-cell-mediated immune responses. Type 2 helper (Th2), Th17, and Th22 cells are involved in both chronic and acute lesions, and Th1 cells are mainly involved in chronic lesions [8].

The skin is the basic immune system responsible for physical and chemical defences [9]. The skin barrier can be divided into two layers—the dermis and epidermis. Keratinocytes are present in the granular layer of the scarf skin and are connected by tight junctions, adherens junctions, and desmosomes. These factors activate keratinocytes, which produce a variety of cytokines and chemokines [10, 11]. In a number of studies performed by various researchers, the proinflammatory cytokines interleukin-6 (IL-6) and interleukin-1*β* (IL-1*β*) induce the proliferation of keratinocytes and activate other immune cells to cause inflammation [12]. Interleukin-8 (IL-8) is known to be a proinflammatory cytokine that induces an immune response through phagocytosis [13, 14]. Overproduction of these inflammatory cytokines is related to the pathophysiology of inflammatory medical disorders such as cancer, cystic fibrosis, rheumatoid arthritis, and cardiovascular disease [15]. Thymus and activation-regulated chemokine (TARC/CCL17), monocyte chemoattractant protein 1 (MCP1/CCL2), and macrophage-derived chemokine (MDC/CCL22) belong to the chemokine (C-C motif) family and participate in inducing immune cell infiltration into inflammatory sites. In particular, they are known to cause Th2 cell migration through the C-C chemokine receptor type 4 (CCR4) gene [1, 16]. AD lesions are characterized by high levels of MDC and TARC [17]. Therefore, MDC and TARC produced by keratinocytes may mediate inflammatory skin conditions such as seborrheic dermatitis, atopic eczema, and AD [18].

*Lagerstroemia ovalifolia* Teijsm. & Binn. (LO) is an evergreen species belonging to the genus *Lagerstroemia* [19]. It mainly grows in Southeast Asia, Malaysia, Cambodia, Vietnam, Laos, Thailand, and Indonesia. The tree has a spreading crown that grows to 15–20 metres or more. Trees are harvested in the wild and used as medicines or as wood. Wood is used for a variety of purposes, such as medium-heavy construction (door and window frames), panels, flooring, and furniture manufacturing. The medicines are used to treat diarrhoea (bark), malaria, and skin diseases (leaves). However, few studies have investigated the mechanisms underlying these effects.

The aim of this study was to identify the effects of natural products that could be used for long-term AD treatment because of their low side effects [20]. Thus, we examined the anti-inflammatory properties of CHCl_3_ fraction number 5 of LO (LOC), which blocks the JAK/STAT1, PI3K/AKT, and NF-*κ*B pathways in tumour necrosis factor-alpha (TNF*α*)/interferon-gamma- (IFN*γ*-) stimulated HaCaT cells.

## 2. Materials and Methods

### 2.1. Plant Material

*Lagerstroemia ovalifolia* Teijsm. & Binn. was collected from Ujung Kulon National Park in the province of Banten, Indonesia. Plant samples were collected and identified by staff at the Center for Pharmaceutical and Medical Technology (PTFM; Tangerang, Indonesia) and verified at the Herbarium Bogoriense (LIPI; Bogor, Indonesia). Voucher specimens recorded as KRIB 0038535 and PMT 537 have been deposited in the herbarium (KRIB) of the Korea Research Institute of Bioscience and Biotechnology (Daejeon, Korea) as well as in the Center for Pharmaceutical and Medical Technology (PTFM) and the Herbarium Bogoriense.

### 2.2. Preparation of Extracts

After drying and grinding *L. ovalifolia*, the powder (10.8 kg) was added to methanol (100 L) at room temperature three times for 1 day to obtain approximately 1.03 kg (9.53%) of solid extract, which was then suspended in water and partitioned with solvents of increasing polarity to generate n-hexane- (100.5 g), CHCl_3_- (73.6 g), EtOAc- (91.9 g), *n*-BuOH- (138.2 g), and water-soluble layers (395.6 g). The CHCl_3_ layer (LOC, 2.0 g) was fractionated on a reversed-phase silica gel column (7 × 50 cm, Zeoprep C18, 45–60 M, Zeochem, Louisville, USA) using Puriflash 450 MPLC (Interchim, Montlucon, France) eluted with MeOH–H_2_O (0–90 min, 0–100% MeOH; 90–135 min, 100% MeOH) to yield seven fractions (LOC Frs. 1–7). All solvents used for column chromatography were of analytical grade (SK Chemicals Co., Ltd., Seongnam-Si, Korea). Evaporation of each solvent under reduced pressure yielded the layers and fractions, which were used for biological activities. Material separation was also completed (data not shown).

### 2.3. Cell Culture

Cells were cultured in 10% foetal bovine serum (FBS; Gibco®, Thermo Fisher Scientific, Inc., Waltham, MA, USA) and 1% penicillin-streptomycin containing Dulbecco's modified Eagle's medium (DMEM; Gibco-BRL, Grand Island, NY, USA). The human keratinocyte HaCaT cell line was purchased from CLS Cell Lines. Service HaCaT cells were maintained at 60–80% density and incubated at 37°C in an atmosphere of 5% CO_2_.

### 2.4. MTT Assay

Cell viability was measured by the 3-(4, 5-dimethylthiazol-2-yl)-2, 5-diphenyl tetrazolium bromide (MTT) assay. Cells were seeded in a 96-well plate (SPL, Gyeonggi-do, Korea) at a concentration of 1 × 10^4^ cells/well. After incubation for 6 h at 37°C, the cells were treated with LOC at a specific concentration. After incubation for 20 h, 5 *µ*l of 5 mg/ml MTT solution (Amresco, LLC, Solon, OH, USA) was added to each well and reacted for an additional 4 h for the formation of formazan. The supernatant was removed, and dimethyl sulfoxide (DMSO) was added to dissolve the formazan. The samples were measured at an absorbance at 570 nm using a microplate reader. The formazan value without the LOC was set to 100%.

### 2.5. ELISA

HaCaT cells were seeded in a 96-well plate at a density of 5 × 10^4^ cells/well and cultured for 6 h. Then, the cells were pretreated with LOC at a specific concentration. After 1 h, the cells were treated with TNF*α*/IFN*γ* (10 ng/ml; TNF*α* Cat. No. 300-01A; IFN*γ* Cat. No. 300-02, PeproTech, Inc., NJ, USA). After 24 h of incubation, the supernatant was harvested. The effect of reducing the production of IL-6 (Cat. No. 555220, BD Biosciences, Santa Clara, CA, USA), IL-8 (Cat. No. DY208, R & D system, Inc., MN, USA), IL-1*β* (Cat. No. 557953, BD Biosciences, Santa Clara, CA, USA), MCP1 (Cat. No. DY279, R & D system, Inc., MN, USA), TARC (Cat. No. DY364, R & D system, Inc., MN, USA), and MDC (Cat. No. DY336, R & D system, Inc., MN, USA) by LOC was measured. The analysis was performed according to the manufacturers' instructions.

### 2.6. Real-Time RT-PCR Analysis

HaCaT cells were cultured by adding LOC and inducer (TNF*α*/IFN*γ*, 10 ng/ml) according to the specific conditions. Total RNA was isolated from the harvested cell pellet using the TRIzol® reagent (Invitrogen, Thermo Fisher Scientific, Inc., MA, USA). RNA was transcribed to cDNA manually with the QuantiTect reverse transcription kit (Cat. No. 205310, Qiagen, Hilden, Germany). Reactions were performed using the SYBR Green PCR Master Mix (KAPA Biosystem, Woburn, Massachusetts) and primers specified in Supplementary Table 1. Reaction products were electrophoresed on a 1.5% agarose gel and stained with the RedSafe™ kit (iNtRON Biotechnology, Inc., Gyeonggi-do, Korea). They were then visualized by ultraviolet light, and the images were stored with an Olympus C4000 zoom camera system (Olympus, Tokyo, Japan).

### 2.7. Western Blot Analysis

After stimulation with an inducer (TNF*α* and IFN*γ*, 10 ng/ml) for 2 h, cells were washed with cold phosphate-buffered saline (PBS) and harvested. The cell pellet was resuspended using lysis buffer (NP40; Cat. No. EBA-1049, ELPIS-Biotech, Inc., Daejeon, Korea), which was supplemented with protease and phosphatase inhibitors. The pellet was kept on ice for 30 min and centrifuged at 20,000 ×g for 10 min at 4°C. The supernatant was stored at −70°C until use in the assay. The protein concentration was measured using the Pierce™ BCA Protein Assay kit (Cat. No. 23225, Thermo Fisher Scientific, Inc., MA, USA). The same amount of protein was separated using 10% sodium dodecyl sulfate-polyacrylamide gels (SDS-PAGE). Protein was transferred to a hydrophilic polyvinylidene fluoride (PVDF) membrane (EMD Millipore, Billerica, MA, USA). The membrane was exposed to 5% skim milk in TBS-T (Tris-buffered saline containing 0.1% Tween 20) to prevent nonspecific binding. The primary antibodies were diluted in the ratio of 1 : 1000 in 5% skim milk, added to the membrane, and incubated overnight at 4°C. The antibodies used are as follows: p65 (Cat. No. #8242, Cell Signaling Technology, Danvers, MA, USA), p-p65 (Cat. No. #3033, Cell Signaling Technology), I*κ*B*α* (Cat. No. #9242, Cell Signaling Technology), p-I*κ*B*α* (Cat. No. #2859, Cell Signaling Technology), JAK1 (Cat. No. sc-376996, Santa Cruz Biotechnology), pJAK1 (Cat. No. #44-422G, Thermo Fisher Scientific, Inc., MA, USA), STAT1 (Cat. No. sc-464, Santa Cruz Biotechnology), pSTAT1 (Cat. No. 9167s, Cell Signaling Technology), PI3K p110 (Cat. No. MA5-15132, Thermo Fisher Scientific, Inc., MA, USA), PI3K p85 (Cat. No. NBP1-51410, Novusbio), AKT (Cat. No. #4691, Cell Signaling Technology), p-AKT (Cat. No. #4060, Cell Signaling Technology), LaminA/C (Cat. No. sc-376248, Santa Cruz Biotechnology), and *β*-actin (Cat. No. #4967, Cell Signaling Technology). After exposure to the horseradish peroxidase- (HRP-) conjugated secondary antibody (antirabbit, Cat. No. sc-2030, Santa Cruz Biotechnology, Inc.; 1 : 5000 dilution; in 5% skim milk), the antibody-specific protein was visualized using the chemiluminescence system (ECL; Cat. No. 32106, Thermo Fisher Scientific, Inc., MA, USA).

### 2.8. Luciferase Assay

The pGL4.32 (luc2P/NF-*κ*B-RE/Hygro) vector (Promega, Madison, WI, USA) was transfected into HaCaT cells using the Lipofectamine® 2000 Reagent (Invitrogen, Thermo Fisher Scientific, Inc., MA, USA). Transfected HaCaT cells (5 × 103 cells/well) were cultured in 96-well plates for 6 h. After the cells were treated with LOC at a specific concentration, inducer TNF*α*/IFN*γ* (10 ng/ml) was added 1 h later. The next day, each well was washed with cold PBS, and the cells were lysed with passive lysis buffer (5х PLB, Cat. No. E1941, Promega, Madison, WI, USA). Each cell lysate treated with LOC was transferred to a white plate. The luciferase assay substrate solution (Luciferase Assay System, Cat. No. E1501, Promega, Madison, WI, USA) was added, and the luciferase activity was measured using a microplate reader (SPARK, Tecan Trading AG, Switzerland).

### 2.9. Immunocytochemistry Analysis

HaCaT cells located on plastic chamber slides (Thermo Fisher Scientific, Inc., MA, USA) were pretreated with LOC for 1 h followed by treatment with the inducer TNF*α*/IFN*γ* (10 ng/ml) for 2 h. After washing with cold PBS, fixation was performed for 10 min using 4% paraformaldehyde in PBS. Next, the cells were treated with 0.1% Triton X-100 (Bio-Rad Laboratories, Inc., WA, USA) in PBS for 10 min and then blocked with 2% bovine serum albumin (BSA) in PBS for 1 h. The primary antibody (NF-*κ*B p65, Cat. No. #8242, Cell Signaling Technology, Inc., MA, USA; 1 : 800 dilution) was diluted in 1% BSA in PBS and incubated overnight at 4°C. Then, Alexa Fluor 488-conjugated secondary antibody (Cat. No. A-11034, Thermo Fisher Scientific, Inc., MA, USA; 1 : 400 dilution) was incubated for 2 h. The nuclei were stained with DAPI (Hoechst 33342, Thermo Fisher Scientific, Inc., MA, USA) diluted in PBS for 5 min. The slide was mounted (Vectashield® medium, Vector Labs, Inc., Burlingame, CA, USA) and visualized with an LSM 510 Meta system (Carl Zeiss AG, Oberkochen, Germany).

### 2.10. Statistical Analysis

For the statistical analysis, values are expressed as the mean ± SEM. Statistical significance was determined using two-tailed Student's t-test for independent means. *P* < 0.05 was considered to indicate a statistically significant difference.

## 3. Results

### 3.1. Anti-Inflammatory Effect of the Active Fraction of LO in TNF*α*/IFN*γ*-Stimulated HaCaT Cells

**Figure 1:**
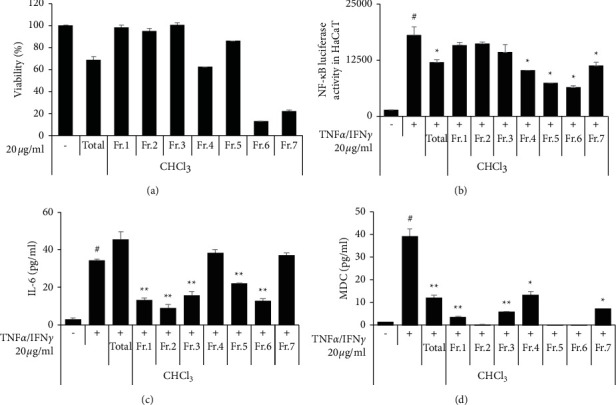
Effect of the CHCl_3_ fraction in TNF*α*/IFN*γ*-stimulated HaCaT cells. (a) HaCaT cells were treated with 20 *µ*g/ml CHCl_3_ fractions, and viability was measured by the MTT assay. (b) HaCaT cells were treated with 20 *µ*g/ml CHCl_3_ fractions, and after 1 h, the cells were stimulated with TNF*α*/IFN*γ* (10 ng/ml). After 16 h, luciferase activity was measured. After stimulation with TNF*α*/IFN*γ*, culture media were harvested 24 h later. (c) IL-6 and (d) MDC ELISA kits were used. The data are presented as the mean ± SEM of three samples. Statistically significant differences (^*∗*^*P* < 0.05 and  ^*∗∗*^*P* < 0.01) between the treatment groups are indicated by different letters.

Previously, we selected the extract of leaf (or bark) LO as an anti-inflammatory resource. Thus, to select the active fraction, we examined the anti-inflammatory activity and cytotoxicity of the fractions. As a result, CHCl_3_ fractions 4, 6, and 7 showed the highest cytotoxicity (Figure 1(a)). IL-6 production was reduced by CHCl_3_ fraction numbers 4 and 7 (Figure 1(c)) but not by the total extract, while MDC production was reduced in all samples (Figure 1(d)). In addition, NF-*κ*B luciferase activity was particularly inhibited by CHCl_3_ fractions 5 and 6 (Figure 1(b)). These results show that the CHCl_3_ fraction has low cytotoxicity and inhibits the production of IL-6 and MDC by inhibiting the NF-*κ*B pathway. Thus, we confirmed the effects of various concentrations of CHCl_3_ fraction number 5 (LOC).

### 3.2. Cytotoxic Effects of LOC in HaCaT Cells

**Figure 2:**
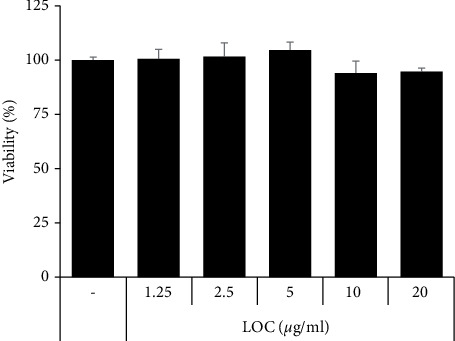
Cytotoxicity of LOC in HaCaT cells. HaCaT cells were incubated in the presence or absence of 0–20 *μ*g/ml LOC, and cell viability was determined using the MTT assay.

Assessment of the biological activity of LOC should be based on the assumption of no deleterious effect on cell metabolism. HaCaT cells were cultured for 24 h with a wide range of concentrations (1.25–20 *µ*g/ml) of LOC. Viability associated with mitochondrial activity was assessed by the MTT assay (Figure 2). Cell viability began to decline slightly at concentrations above 20 *µ*g/ml. Subsequent experiments were performed in a range of concentrations (2.5–20 *µ*g/ml) where cell viability was thought to be stable.

### 3.3. LOC Inhibits the Production of Proinflammatory Cytokines and Chemokines in TNF*α*/IFN*γ*-Stimulated HaCaT Cells

**Figure 3:**
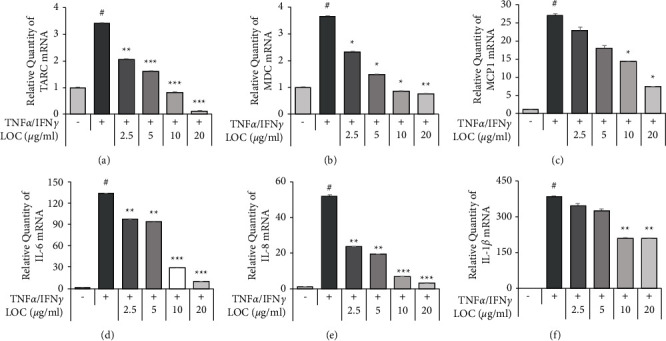
LOC reduces proinflammatory cytokine production in TNF*α*/IFN*γ*-stimulated HaCaT cells. HaCaT cells were pretreated with LOC (2.5, 5, 10, and 20 *µ*g/ml) for 1 h and then stimulated with TNF*α*/IFN*γ* (10 ng/ml). Total RNA was isolated, and the mRNA levels of TARC (a), MDC (b), MCP1 (c), IL-6 (d), IL-8 (e), and IL-1*β* (f) were measured by real-time PCR. The data are presented as the mean ± SEM of three samples. Statistically significant differences (^*∗*^*P* < 0.05) between the treatment groups are indicated by different letters.

Cytokines and chemokines increase as the inflammatory reaction progresses; therefore, they were measured as indicators. CCL2 (MCP1), TARC, and MDC are characterized by high expression levels in atopic lesions. This group of inflammatory cytokines is the most important group of compounds participating in the pathogenesis of inflammation [21]. Among the many representatives of this group, the greatest importance is attributed to IL-1*β*, IL-6, IL-8, and so on. Therefore, the protein products of TARC, MDC, MCP1, IL-1*β*, IL-6, and IL-8 contained in the media of HaCaT cells treated with various concentrations of LOC were investigated. The TARC, MDC, MCP1, IL-1*β*, IL-6, and IL-8 mRNA levels were measured using real-time PCR. Additionally, their mRNA levels were measured by RT-PCR, which showed that the mRNA levels were inhibited more than the protein levels (Figure 3).

### 3.4. LOC Inhibits the Production of Chemokines in TNF*α*/IFN*γ*-Stimulated HaCaT Cells

**Figure 4:**
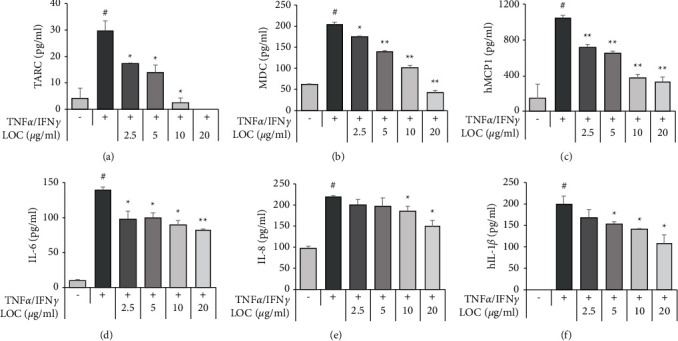
LOC reduces chemokine production in TNF*α*/IFN*γ*-stimulated HaCaT cells. HaCaT cells were pretreated with LOC (2.5, 5, 10, and 20 *µ*g/ml) for 1 h and then stimulated with TNF*α*/IFN*γ* (10 ng/ml). After 24 h, culture media were harvested. LOC and TNF*α*/IFN*γ* treatment was performed under the same conditions, and cell incubation was carried out for 24 h. The protein levels of TARC (a), MDC (b), MCP1 (c), IL-6 (d), IL-8 (e), and IL-1*β* (f) were measured by ELISA. The data are presented as the mean ± SEM of three samples. Statistically significant differences (^*∗*^*P* < 0.05 and  ^*∗∗*^*P* < 0.01) between the treatment groups are indicated by different letters.

Th2 chemokines typically induce Th2 cell migration and infiltration into inflammatory sites. TNF*α*/IFN*γ* is a representative inducer known to induce various chemokines or cytokines in HaCaT cells. On the other hand, LOC attenuated the increased TARC, MDC, MCP1, IL-1*β*, IL-6, and IL-8 products in a dose-dependent manner, as shown by ELISA. The TARC, MDC, MCP1, IL-1*β*, IL-6, and IL-8 proteins were measured using specific ELISA kits (Figure 4).

### 3.5. LOC Inhibits the Transcriptional Activity of NF-*κ*B in TNF*α*/IFN*γ*-Stimulated HaCaT Cells

**Figure 5:**
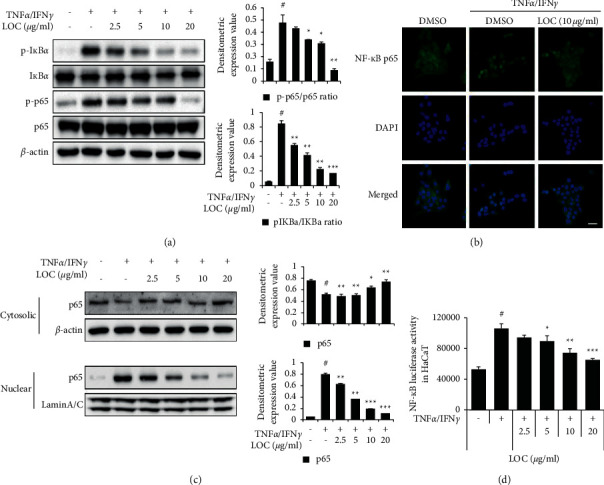
LOC reduces NF-*κ*B activation and translocation. (a, c) HaCaT cells were pretreated with LOC for 1 h and then stimulated with TNF*α*/IFN*γ* for 2 h. The phosphorylation levels of the indicated proteins were determined in whole-cell lysates by western blotting. (b) The translocation of NF-*κ*B p65 was confirmed through immunocytochemistry as described in the Section 2. (d) Luciferase activity was measured in the presence of LOC. LOC (2.5, 5, 10, and 20 *µ*g/ml) was processed, and after 1 h, TNF*α*/IFN*γ* (10 ng/ml) was added. After 16 h, luciferase activity was measured. The data are presented as the mean ± SEM of three samples. Statistically significant differences (^*∗*^*P* < 0.05 and  ^*∗∗*^*P* < 0.01) between the treatment groups are indicated by different letters.

NF-*κ*B is activated by phosphorylation and has a role as a transcription factor that regulates the expression of cytokines and chemokines, such as IL-6, IL-8, TARC, and MDC. I*κ*B*α* initiates the activation of NF-*κ*B by phosphorylation. In our experiment, the decrease in phosphorylated I*κ*B*α* and NF-*κ*B subunit p65 protein in the LOC-treated group was confirmed by western blot (Figures 5(a) and 5(c)), indicating that activation of NF-*κ*B signalling was decreased. *β*-Actin and LaminA/C were used as internal controls. We also visualized the NF-*κ*B subunit p65 by immunocytochemistry and confirmed that its translocation was reduced by LOC (Figure 5(b)).Additionally, luciferase analysis confirmed that LOC decreased the binding of the NF-*κ*B transcription factor at the promoter region (Figure 5(d)). The results show that LOC inhibits the phosphorylation of the NF-*κ*B subunit p65 and its translocation from the cytosol to the nucleus in HaCaT cells.

### 3.6. LOC Inhibits the Phosphorylation of JAK/STAT and PI3K/AKT in TNF*α*/IFN*γ*-Stimulated HaCaT Cells

**Figure 6:**
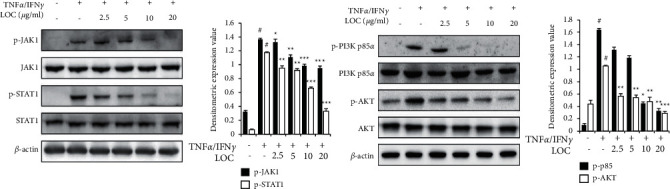
Inhibitory effects of LOC on the JAK/STAT1 and PI3K/AKT pathways. HaCaT cells were preincubated with LOC for 1 h and then treated with TNF*α*/IFN*γ* for 2 h. The p-JAK, pSTAT1, PI3K p-p85, and p-AKT protein levels were determined with an immunoblot assay. The data are presented as the mean ± SEM of three samples. Statistically significant differences (^*∗*^*P* < 0.05 and  ^*∗∗*^*P* < 0.01) between the treatment groups are indicated by different letters.

The JAK/STAT pathway is important to the immune system, and interferons stimulate the JAK/STAT pathway. Activated JAKs subsequently phosphorylate additional targets, including both the receptors and major substrates of STATs. STATs are latent transcription factors that reside in the cytoplasm until activation. Thus, the JAK/STAT1 cascade provides a direct mechanism to translate an extracellular signal into a transcriptional response [22]. The PI3K pathway is activated by IFN*γ*, and PI3K is known to be activated by IFN*α*/*β*. PI3K activation is required for the induction of STAT1 serine phosphorylation in response to IFN*γ* and for full activation of gene expression [23]. To investigate whether LOC treatment resulted in the downregulation of TNF*α*/IFN*γ*-mediated JAK/STAT and PI3K/AKT activation in HaCaT cells, western blot analysis using specific antibodies was utilized (Figure 6). LOC pretreatment was applied 1 h prior to TNF*α*/IFN*γ* exposure in HaCaT cells. TNF*α*/IFN*γ* increased the phosphorylation of JAK/STAT1 and PI3K/AKT for 1 h. As a result, LOC was revealed to inhibit the phosphorylation of the JAK/STAT1 pathway and PI3K/AKT pathway in TNF*α*/IFN*γ*-stimulated HaCaT cells (Figure 6). These results revealed that LOC inhibited the activation of JAK/STAT and PI3K/AKT in HaCaT cells.

## 4. Discussion

Because the world population is constantly ageing, lifestyle diseases and chronic diseases have become more prevalent, and more people are pursuing an improved quality of life by focusing on preventing diseases. Accordingly, drug development has shifted its focus towards treating ageing, cancer, chronic diseases, and lifestyle-related diseases [24].

Previous studies have confirmed the anti-inflammatory activity of LO in lipopolysaccharide- (LPS-) stimulated RAW264.7 macrophage cells [19]. LO was confirmed to decrease the levels of nitric oxide (NO), inducible nitric oxide synthase (iNOS), cyclooxygenase-2 (COX- 2), prostaglandin E2 (PGE2), and various inflammatory cytokines by inhibiting the NF-*κ*B pathway [19]. Therefore, the anti-inflammatory effect of LOC was studied in detail in TNF*α*/IFN*γ*-stimulated HaCaT cells. In the present study, LOC inhibited inflammatory cytokines and chemokines in TNF*α*/IFN*γ*-stimulated HaCaT cells in a dose-dependent manner but had no effect on cell viability (Figure 2). As a result, we determined that LOC was effective on TNF*α*/IFN*γ*-stimulated HaCaT cells in atopic inflammation. Inflammation regulates immune responses and the pathogenesis of a number of diseases. This process is controlled by inflammatory mediators such as cytokines and chemokines [25]. In this study, LOC significantly inhibited the production of IL-6, IL-8, IL-1*β*, MCP-1, TARC, and MDC. IL-6 and IL-8 are inflammatory cytokines that mediate the immune response. These cytokines were found to be increased in keratinocytes stimulated by TNF*α*/IFN*γ*. However, cytokine production was inhibited at the protein and RNA levels by the biological activity of LOC (Figure 3). In addition, stimulated keratinocytes produce chemokines such as TARC and MDC and regulate the activation of normal T cell expressed and secreted (RANTES/CCL5). These chemokines have an important role in the pathogenesis of AD [26, 27]. For example, chemokines differentiate T lymphocytes and recruit leukocytes to sites of inflammation [28, 29]. In this study, TARC and MDC were increased by TNF*α*/IFN*γ* but significantly decreased by LOC (Figure 4). These results indicated that LOC exerted an ameliorative effect on TNF*α*/IFN*γ*-induced keratinocyte influx and the release of inflammatory molecules.

**Figure 7:**
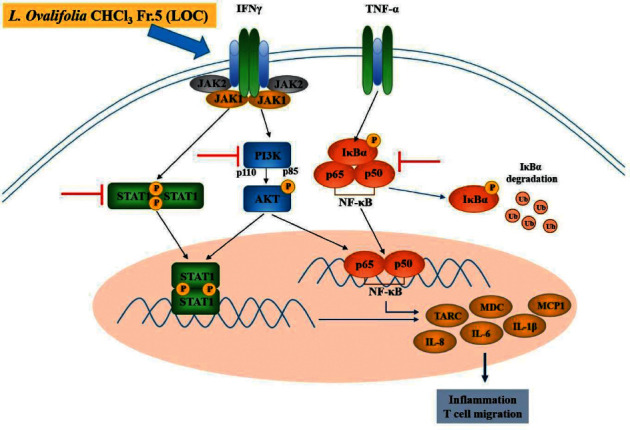
Schematic diagram illustrating the mechanisms underlying the anti-inflammatory effects of LOC. LOC, *Lagerstroemia ovalifolia* CHCl_3_ fraction 5 extract; TNF*α*, tumour necrosis factor-*α*; IFN*γ*, interferon gamma; NF-*κ*B, nuclear factor-*κ*B; I*κ*B*α*, inhibitor of NF-*κ*B; Ub, ubiquitination; JAK1, Janus kinase 1; JAK2, Janus kinase 2; STAT1, signal transducer and activator of transcription 1; PI3K, phosphoinositide 3-kinase; AKT, protein kinase B (PKB); TARC/CCL17, thymus and activation-regulated chemokine; MDC/CCL22, macrophage-derived chemokine; IL-8, interleukin-8; IL-1*β*, interleukin-1 beta; IL-6, interleukin-6; and MCP-1, monocyte chemoattractant protein-1.

NF-*κ*B has an important role in regulating the expression of inflammatory mediators in immune and inflammatory responses and is induced by stimuli such as LPS, TNF*α*, and IFN*γ* [30, 31]. These stimuli cause events including phosphorylation, ubiquitination, and degradation through binding to receptors [25, 32]. The phosphorylation of I*κ*B leads to the loss of the inactivating function of NF-*κ*B. As a result, NF-*κ*B is activated and translocates into the nucleus and then functions as a transcription factor. Our results show the downregulation of NF-*κ*B through the inhibition of phosphorylation and translocation by LOC (Figure 5). In addition, JAK/STAT1 and PI3K/AKT were phosphorylated by stimulation. NF-*κ*B is activated and expected to induce inflammation because the relationship between JAK/STAT1, PI3K/AKT, and NF-*κ*B has already been studied. NF-*κ*B is activated through the phosphorylation of I*κ*B kinases (IKKs) by phosphorylated PI3K/AKT [33–35]. Our results show that LOC inhibits the phosphorylation of JAK/STAT1 and PI3K/AKT (Figure 6), which is expected to prevent the activation of NF-*κ*B (Figure 5). The inactivation of NF-*κ*B, JAK/STAT1, and PI3K/AKT by LOC can inhibit inflammation, but more research on the mechanisms is needed (Figure 6). These results suggested that the underlying molecular mechanisms of LOC are closely associated with the downregulation of PI3K/AKT and NF-*κ*B activation. The present study observed the effects of LOC on JAK/STAT1, PI3K/AKT, and NF-*κ*B, which are involved in anti-inflammatory responses. LOC inhibited JAK/STAT1, PI3K/AKT, and NF-*κ*B phosphorylation through TNF*α* and IFN*γ* stimulation in HaCaT cells. The results of the present study indicated that LOC has anti-inflammatory and antiatopic dermatitis effects via various molecular targets by directly or indirectly acting on signalling pathways stimulated by TNF*α*/IFN*γ* (Figure 7).

In summary, LOC inhibited inflammation by blocking JAK/STAT1, PI3K/AKT, and NF-*κ*B activation in TNF*α*/IFN*γ*-stimulated HaCaT cells (Figure 5). The inhibition of AKT phosphorylation may have reduced the activation of IKKs. In addition, LOC-mediated inhibition of I*κ*B*α* and p65 phosphorylation decreased NF-*κ*B activation (Figure 5), which was also confirmed by luciferase activity. In addition, LOC was confirmed to inhibit JAK/STAT1 phosphorylation by IFN*γ*, implying that LOC has an anti-inflammatory activity (Figure 6). As a result, the production of IL-6, IL-8, IL-1*β*, MCP1, TARC, and MDC was reduced by LOC (Figures 3 and 4). The inhibition of inflammation and T-cell migration can be expected by controlling inflammatory cytokines and chemokines. Therefore, LOC can be used as a therapeutic agent for inflammatory skin diseases.

## 5. Conclusions

In conclusion, the present study confirmed that LOC inhibits the expression of AD-associated chemokines and cytokines in TNF*α*/IFN*γ*-induced keratinocytes. These effects were considered to be associated with the suppression of JAK/STAT1, PI3K/AKT, and NF-*κ*B activation. These experimental results provide a scientific basis for the use of LOC to treat AD. Additional experiments to test the in vitro effects of LOC on AD are currently in progress in our laboratory.

## Data Availability

All data generated or analyzed during this study are included in this published article.

## Supplementary Materials

Supplementary Materials Supplementary Table 1: primers used for real-time PCR.
