# Supplementary material for: Anti-Inflammatory Effects of Lagerstroemia ovalifolia Teijsm. & Binn. in TNFα/IFNγ-Stimulated Keratinocytes
Source: Evid Based Complement Alternat Med. 2021 Nov 9;2021:2439231. doi: 10.1155/2021/2439231 (PMC8594990; doi:10.1155/2021/2439231)
Supplement: Supplementary Materials — Supplementary Table 1: primers used for real-time PCR. [file 2439231.f1.pdf]

Supplementary Table 1.
Primer used for Real-time PCR

	forward	reverse
**TARC**	CAC GCA GCT CGA GGG ACC AAT GTG	TCA AGA CCT CTC AAG GCT TTG CAG G
**MDC**	AGG ACA GAG CAT GGC TCG CCT ACA GA	TAA TGG CAG GGA GGT AGG GCT CCT GA
**IL-8**	ATG ACT TCC AAG CTG GCC GTG GCT	TTA TGA ATT CTC AGC CCT CTT CAA AAA
**IL-6**	GAC AGC CAC TCA CCT CTT CA	AGT GCC TCT TTG CTG CTT TC
**MCP1**	TCT GTG CCT GCT GCT CAT AG	CAG ATC TCC TTG GCC ACA AT
**IL-1β**	GGA CAA GCT GAG GAA GAT GC	TCT TTC AAC ACG CAG GAC AG
**gapDH**	CCC TCC AAA ATC AAG TGG	CCA TCC ACA GTC TTC TGG
